# Assistant Medical Officers in Asylums

**Published:** 1913-03-01

**Authors:** 


### ASSISTANT MEDICAL OFFICERS IN ASYLUMS.

### The Need for Higher Emoluments and Better Accommodation.

The Medico-Psychological Association of Great Britain and Ireland held their February quarterly meeting on the 20th ult. at Darenth Industrial Colony, Kent, under the presidency of Dr. J. G. Soutar.

Among the subjects discussed was that of the position and prospects of assistant medical officers in asylums. This was introduced in a careful paper by one of the senior members of the Association, Dr. J. Beveridge Spence. He said the title would be familiar to recent readers of the *British Medical Journal*, the letters in which were characterised by moderation of language in setting forth the case in which the writers were interested. He had long felt that something should be done to improve the position of assistant medical officers of asylums, and so he had found himself in accord with the work of the committee of that Association, which had recently issued an interim report on the matter. That report advocated the more advanced education of all candidates for the post of assistant medical officer; indeed, some went so far as to urge that no appointments, certainly no senior appointments, should be given except to holders of the special qualification in mental science. Dr. McDowall had done much to press upon asylum committees the advisability of granting furlough leave to junior members of the medical staff of their asylums, so that they might specially study the subjects required by the universities granting diplomas in psychological medicine. Still, there were doubts as to its practicability, and the money factor must enter into the question. He believed that, with so many new appointments being opened up, committees of asylums would be compelled, by the law of supply and demand, to increase the stipend of the junior medical officers so as to induce the right type of men to enter the speciality. In these days, when tuberculosis officers, school attendance officers, deputy medical officers of health were opening out lucrative careers to young medical men, it daily became more difficult to obtain men whom asylum officers would like to have as colleagues; and when one saw posts advertised with salaries of £400 to £500 a year, with more freedom than assistant medical officers of asylums could hope for, it could not be wondered at that young men should be attracted to them. He, Dr. Spence, considered that a man could not do better than hold a post in an asylum for a few years, provided it were made financially worth his while; for it provided for him experience which he would not get elsewhere, and of a kind which would be useful to him in his subsequent career. What, in a general way, had the junior in the asylum to look forward to? He might receive an advance of salary, but that was not a sufficient inducement to him to stand fast; and many men had grown old in the service without having received a chance of promotion in their own county, or to a higher post elsewhere, simply because they were the victims of circumstances beyond their control. In order to obtain reliable information on the subject, Dr. Spence sent out forms of questions to the superintendents of ninety-four asylums, and ninety-two of them had courteously replied. The number of assistant medical officers concerned was 253. The average minimum salary of senior assistant medical officers was £224, the average maximum £309 (exclusive of emoluments), the lowest being £150, rising to £200; and the highest £330, with a maximum of £430. The medical officers junior to the first seemed to vary from an average minimum of £160 to an average maximum of £208, the highest being £185, rising to £330, and the lowest £130, rising to £150. The emoluments he found difficult to tabulate. As to whether the medical superintendents throughout the country approved of their senior assistants being married, in nearly every case the reply had been in the affirmative. Sixty-seven favoured it without reservation; six provided there was sufficient accommodation; nine thought it should be so in large asylums; while eight regarded the proposal unfavourably. In most instances there was a feeling against the second assistant being permitted to enter the married state. The paper then went on to deal with the question of the provision of quarters for the married assistant officers inside or outside the institution, and gave the figures which he had collected. Comparison of asylum and prison services in the matter of the assistant medical officerships was in favour of the prison officer, for there were eight such posts in which the minimum salary was £400, rising by £15 increments to £550, while six received £300 per annum minimum, increasing by £10 a year to £390. All had quarters allotted to them, or an allowance in lieu thereof, but no board. The well-known sympathetic hearing given by the Commissioners in Lunacy on all matters of asylum administration led him to hope much from a clear statement of the present position of the assistant medical officer; and he regarded it as one of the legitimate objects of the Medico-Psychological Association to assist its assistant medical officer members as far as they reasonably could, just as it had done great work for the betterment of the asylum nurse, to the benefit of the whole service.

Dr. Wolseley Lewis considered that the great difficulty from the medical superintendent's point of view was the question of accommodation; for in most county asylums there was no accommodation such as was contemplated in the paper just read.

Miss Johnson (Virginia Water) voiced the need felt by assistant medical officers to study at some centre for special diplomas, and rub shoulders and compare notes with others having similar aims. Even a week or two added to the ordinary summer vacation would be a help in that direction.

Dr. Sargeant (Eastbourne), who said he had been assistant medical officer at a private asylum, remarked that the Commissioners seldom inquired about the conditions under which the assistant medical officers lived when they paid their visits, though nearly everything else seemed the object of their care and thought; yet there were several ways in which the lot of that officer could be made better.

Dr. Powell considered that the great hardship suffered by assistant medical officers of asylums was the slowness of promotion, and that tempted one to live an unambitious existence. Moreover, there were such men as "chronic assistants"—men who were never likely to rise to anything beyond; and it would be a friendly act to advise such, early in their career, to take up some other work.

Dr. Passmore was strongly of opinion that if the assistant medical officer was required to possess all the special qualifications which were being projected, he should be adequately remunerated. Certainly if a man wished to get married he should not be condemned to celibacy by the conditions imposed upon him.

Dr. Spence briefly replied, demurring to the contention that the Commissioners were not solicitous about the conditions of the assistant medical officers; that was contrary to his experience.

